# How Could We Influence Systemic Inflammation in Allergic Rhinitis? The Role of H1 Antihistamines

**DOI:** 10.1155/2018/3718437

**Published:** 2018-06-12

**Authors:** Ioana Adriana Muntean, Ioana Corina Bocsan, Nicolae Miron, Anca Dana Buzoianu, Diana Deleanu

**Affiliations:** ^1^Department of Allergology and Immunology, “Iuliu Hatieganu” University of Medicine and Pharmacy, Cluj Napoca, Romania; ^2^Department of Pharmacology, Toxicology and Clinical Pharmacology, “Iuliu Hatieganu” University of Medicine and Pharmacy, Cluj Napoca, Romania; ^3^Department of Clinical Immunology, Sahlgrenska University Hospital, Göteborg, Sweden

## Abstract

The aim of the study was the analysis of adhesion molecules' profile (ICAM-1, VCAM-1, and E-selectin) in patients with allergic rhinitis and the influence of H1 antihistamines on those markers. Seventy-nine patients with persistent allergic rhinitis (PAR) and 30 healthy volunteers were included in the study. The patients with PAR were treated with desloratadine 5 mg/day or levocetirizine 5 mg/day for 4 weeks. The clinical (rhinitis symptoms and total symptoms score (TSS), type of sensitization) and biological evaluation (total IgE, eosinophils, ICAM-1, VCAM-1, and E-selectin) as well as fractionate nitric oxide in exhaled air (FeNO) measurement was performed before and after treatment. The plasmatic levels of ICAM-1, VCAM-1, total IgE, and eosinophils and FeNO were significantly increased in patients with PAR compared to healthy volunteers. H1 antihistamines significantly improved TSS, with no differences between the investigated drugs. There was a significant decrease of eosinophils, total IgE, and FeNO after treatment. H1 antihistamines significantly decreased the plasmatic levels of ICAM-1 and E-selectin but not VCAM-1 compared to basal values. There is no difference between levocetirizine and desloratadine in the reduction of CAMs. A systemic inflammation characterized by increased levels of CAMs is present in patients with PAR. H1 antihistamines improve symptoms and reduce CAMs and FeNO levels after 1 month of treatment. H1 antihistamines might reduce the systemic inflammation which could be responsible to asthma occurrence in patients with PAR.

## 1. Introduction

Allergic rhinitis (AR) is an IgE-mediated immune response characterized by an inflammatory process of the nasal mucosa [1]. Now, allergic rhinitis is considered the most prevalent clinical manifestation of allergy, affecting 20–30% of the general population worldwide [1, 2]. AR is also a risk factor for asthma's occurrence; more than 25% of patients with persistent allergic rhinitis (PAR) may develop asthma over time [3].

The immune response to allergen exposure involves several cells and mediators. Immediately after allergen exposure, in the early phase of allergic inflammation there is an immediate release of mast cell products, including histamine. The released mediators generate a specific inflammatory network, which favours the expression and activation of certain cellular adhesion molecules (CAM) [4, 5]. The activation of CAMs favours the migration of proinflammatory cells such as eosinophils and neutrophils in the nasal mucosa [5, 6]. Late-phase immune response is characterized by release of various cytokines, chemokines, and other mediators, mainly produced by TH2 cells and granulocytes, which changes cellular components, with a predominant influx of TH2 cells and eosinophils [5, 6].

Vascular cell adhesion molecule 1 (VCAM-1) and intercellular cell adhesion molecule 1 (ICAM-1) belong to the immunoglobulin superfamily. Both are expressed mainly on endothelial cells [7, 8]. Proinflammatory cytokines like IL-1 and TNF-*α* enhance the expression of both CAMs, while Th2 cytokines significantly enhance VCAM-1 expression [9]. ICAM-1 and VCAM-1 are involved in transendothelial migration and adhesion of leukocytes, including eosinophils [6, 10], contributing in the maintenance of late immune response in the nasal mucosa.

E-selectin is a CAM expressed on the endothelial cell, mediating the rapid low-affinity adhesion of leukocytes to endothelial cells. The level of E-selectin is higher in the early stage of inflammation in the vascular endothelium [8, 9]. E-selectin is an important CAM in the initiation and organization of allergic inflammation.

H1 antihistamines are the first therapeutic option in all forms of allergic rhinitis [1]. Their main effect is related to blockade of H1 receptors, mediating their antiallergic action. Further research found that the new-generation H1 antihistamines have also an anti-inflammatory effect, decreasing the number of inflammatory cells recruited in the tissue and diminishing the expression of CAMs [11–15].

The aim of the study was the analysis of CAMs' evolution under 1-month treatment with levocetirizine and desloratadine, two H1 antihistamines from second generation in patients with PAR under continuous natural exposure to allergens. Secondarily, we also characterized the plasmatic levels of CAMs (ICAM-1, VCAM-1, and E-selectin) in patients with PAR.

## 2. Material and Method

### 2.1. Patients and Clinical Evaluation

In the present study, we performed a post hoc analysis of an initial randomized control trial (RCT) that included patients with PAR and healthy volunteers. The analyzed inflammatory markers represented secondary outcomes of the initial study [16]. Seventy-nine patients with PAR (mean age 30.44 ± 9.9 years and sex ratio M : F = 1.02) were included in the experimental group, while 30 healthy volunteers (mean age 28.92 ± 8.91 years and sex ratio M : F = 1) were included in the control one. The study protocol, inclusion and exclusion criteria, and clinical evaluation were similar to the initial study [16]. The protocol was approved by the Ethics Committee of the “Iuliu Hatieganu” University of Medicine and Pharmacy, Cluj-Napoca. All patients signed the informed consent at enrollment.

The diagnosis of AR was done according to international guidelines, based on history and skin prick test (SPT) [1]. The following demographic data were noticed from anamnesis: age, sex, and living area (rural/urban). The severity of AR was established based on severity of specific symptoms: rhinorrhea, nasal congestion, sneezing, and nasal and ocular itching. The severity was analyzed on a scale from 0 to 3 (0 = absent, 1 = mild, 2 = moderate, and 3 = severe), retrospectively, for 12 hours prior to presentation. The total symptom score (TSS) was calculated by adding the score for every symptom. A TSS < 6 means a mild rhinitis, while a TSS > 6 represents a moderate-severe form of disease.

After the baseline evaluation, the patients were randomly divided into two groups using an adaptive biased-coin randomization. The first group included 39 patients, and they received levocetirizine 5 mg/day, while the second group of 40 patients received desloratadine 5 mg/day. The treatment was recommended for 4 weeks. At the end of the four weeks, the patients were similarly evaluated.

### 2.2. Skin Prick Test (SPT)

The diagnosis of allergy was established through skin prick test, according to international guidelines [17]. The allergen panel included international recommendation and particularities of exposure to allergens in Romania: house dust mites (*Derm. pteronyssinus* and *Derm. farinae*), grass pollens (mixed grasses), cereal pollens (cereals), Betulaceae pollens (spring trees), cat and dog epithelia, *Alternaria alternata*, and weed pollen (*Artemisia vulgaris* and *Ambrosia elatior*). Standardized allergen extracts (Hal Allergy, Netherlands) were used. SPT was done at the beginning of the study.

### 2.3. FeNO Measurement

The measurement of fractionated exhaled nitric oxide (FeNO) was done in accordance to international recommendations [18], using NIOX MINO® (Aerocrine, Sweden). FeNO was measured before and after 1 month of treatment with H1 antihistamines. The measured values were expressed in parts per billion (ppb). A standardized cutoff value of 25 ppb was considered a normal upper limit.

### 2.4. Biological Evaluation

All the biological parameters were determined before and after 1 month of treatment with H1 antihistamines. Total IgE plasmatic level was done using electrochemiluminescence immunoassay method (ECLIA). The obtained values were expressed as UI/ml. A value below 100 UI/ml was considered normal.

The eosinophils (Eo) were manually counted from the peripheral blood on a slide, and their value was expressed as %. We considered a normal value between 2–4%.

The plasmatic levels of ICAM-1, VCAM-1, and E-selectin were determined using ELISA technique (Quantikinine R&D system, USA). The blood sample of 5 ml without anticoagulant was collected and centrifuged within the 1st hour, followed by serum separation. The serum was then stored at −80°C until the determination was performed. All the aforementioned determinations were done according to the manufacturers' instructions. For each assay, samples were diluted as needed, and protein levels were calculated based on four-parameter logistic (4-PL) curve fit.

### 2.5. Statistical Analysis

The statistical analysis was performed using SPSS version 21 (Chicago, IL, USA). Data were labeled as nominal and expressed as percentage and continuous variables. The normal distribution for continuous variables was done using Kolmogorov-Smirnov test. Variables with normal distribution were expressed as mean and standard deviation, while variables with abnormal distribution were expressed as median and 25–75 percentiles.

The adequate statistic tests according to data distribution were chosen. The differences were assessed within groups by Wilcoxon signed-rank test and between groups by Mann–Whitney test. The influence of different parameters on CAM evolution in time was done using ANOVA test for repeating measurements. The Spearman coefficient of correlation was calculated to highlight differences between continuous variables. The level of statistical significance was set at *p* < 0.05.

## 3. Results

Patients' demographic data are presented in Table 1.

### 3.1. Initial Evaluation

Fifty-six (70.9%) patients presented persistent moderate-severe forms of AR. The initial TSS proved the severity of AR (median 8 (5–11)). Forty-one patients (51.9%) had multiple sensitizations to both indoor and outdoor allergens. The basal TSS was not correlated with the duration of AR but was significantly higher in patients with sensitization to pollen or multiple sensitizations (*p* = 0.01).

In patients with AR, plasmatic ICAM-1 and VCAM-1 were significantly increased compared to healthy volunteers (*p* < 0.001 and *p* < 0.001, resp.), with no differences between the groups. E-selectin was similar in healthy volunteers and patients with AR (Table 2).

The severity of AR expressed as a high value of TSS was correlated with the plasmatic level of E-selectin (*R* = 0.996, *p* < 0.001), but not with basal levels of ICAM-1 (*R* = −0.051, *p* = 0.657) or VCAM-1 (*R* = −0.056, *p* = 0.622). There is no correlation between the plasmatic level of CAM and patients' age, sex, or type of sensitization (*p* > 0.05). There is a positive correlation between the basal values of ICAM-1 and E-selectin (*R* = 0.353, *p* = 0.001).

Total IgE and Eo were significantly increased at baseline, with no differences between the groups (*p* = 0.408 and *p* = 0.838, resp.). The initial value of peripheral Eo was strongly correlated with total IgE (*R* = 0.853; *p* < 0.001). There was no correlation between basal Eo or total IgE levels and ICM-1, VCAM-1, and E-selectin (*p* > 0.05).

FeNO was increased in patients with AR (median 27 (18–46)) compared to the standardized cutoff value (25 ppb) (*p* < 0.001). There was no difference between the groups regarding the basal value of FeNO. There was no correlation between initial FeNO and severity of rhinitis' symptoms, type of sensitization, or basal values of CAM (*p* > 0.05).

### 3.2. One-Month Evaluation

H1 antihistamines significantly improved all the symptoms after 1 month of treatment. TSS significantly decreased after treatment (median 8 (5–11) versus median 0 (0–4), *p* = 0.01), with no differences between the investigated drugs (*p* = 0.571).

1-month evaluation revealed a significant decrease of IgE plasmatic level (*p* < 0.001), especially in patients with monosensitization either to indoor or outdoor allergens (*p* = 0.05). The reduction of total IgE was not influenced by the type of treatment; patients' age, sex, and environment; or duration of AR (*p* > 0.05) (Table 3). Total IgE significantly decreased in patients with moderate-severe forms of AR compared to those with mild disease (*p* = 0.05).

Same pattern was noticed also for peripheral Eo, with a significant reduction after treatment (*p* = 0.04). The Eo significantly decreased after 1 month of treatment especially in patients with monosensitization to indoor allergens or mixed sensitization (*p* = 0.002). Eo reduction was also significant in patients with severe forms of AR (*p* = 0.04). The reduction of Eo was not influenced by the type of treatment; patients' age, sex, and environment; or duration of AR (*p* > 0.05).

After the four-week treatment, H1 antihistamines significantly decreased the plasmatic levels of ICAM-1 (*p* = 0.049) and E-selectin (*p* = 0.002), but not VCAM-1 (*p* = 0.310) compared to basal values. There was no difference between levocetirizine and desloratadine in the reduction of adhesion molecule plasmatic levels (Table.  3). We noticed a significant reduction of CAM levels in patients with moderate-severe forms compared to patients with mild rhinitis (VCAM-1 *p* = 0.037, ICAM-1 *p* = 0.001, and E-selectin *p* = 0.002). The reduction of CAM levels was not influenced by patients' age, sex, and type of sensitization.We also analyzed the improvement of symptoms in correlation with inflammatory markers. The reduction of TSS was positively correlated with the reduction of ICAM-1 (*R* = 0.238, *p* = 0.035), but it was not correlated with VCAM-1 and E-selectin evolutions. ICAM-1 reduction was positively correlated with E-selectin (*R* = 0.504, *p* < 0.001) and VCAM-1 (*R* = 0.711, *p* < 0.001) evolutions.

FeNO was significantly reduced after 1-month treatment with AH1, desloratadine being more effective than levocetirizine (Table 3). The reduction was not influenced by patients' age, severity of allergic rhinitis or number of sensitization (*p* > 0.05), or the type of it (*p* > 0.05). FeNO had a more significant reduction in male compared to female patients (*p* = 0.036). The reduction of FeNO did not correlate with basal plasmatic levels of CAMs. The reduction of FeNO was not correlated with symptoms' improvement. The reduction of FeNO was minimal in patients sensitized to pollen compared with patients with multiple sensitization or with sensitization to indoor allergens, but the difference did not reach the level of statistical significance (median −6 (−32 to −3) versus median −12 (−35 to −1.5) versus median −11 (−35 to −2), *p* > 0.05).

## 4. Discussion

This study assessed the effect of H1 antihistamines from the 2nd generation, showing that both levocetirizine and desloratadine improved symptoms and reduced the level of inflammation in allergic rhinitis. We also characterized the plasmatic profile of adhesion molecules in patients with persistent allergic rhinitis.

AR is characterized by the presence of inflammation in the nasal mucosa. The exposure to allergens mediates the release of mediators from mast cells, especially histamine, which are responsible for the characteristic symptoms of AR (sneezing, nasal itching, and rhinorrhea) [19]. But these mediators will also stimulate infiltration of the nasal mucosa with inflammatory cells, including eosinophils [20]. The chronic inflammatory response with eosinophil infiltration in the nasal mucosa is the pattern of allergic inflammation [1, 19]. These cells continue to produce cytokines, chemokines, and other inflammatory mediators, which leads to persistent symptoms and tissue structural changes and damages. Thus, rhinitis progression and persistence become more dependent on mediators which promote infiltration of cells, such as eosinophils and TH lymphocytes [21]. AR is a risk factor for asthma development and may appear before or after asthma onset. Allergic inflammation is the key to understand both diseases and the mechanisms of rhinitis progression to asthma [5, 19].

The eosinophils migrate at the inflammation site due to the high expression of the adhesion molecules on the endothelial cell surfaces [22]. The role of adhesion molecules in the pathogenesis of allergic diseases was investigated in many studies [10, 23–29]. Most of them showed an increase of ICAM-1 and VCAM-1 in nasal lavage fluid, mucosa biopsies, and serum in patients with AR versus healthy subjects, after allergen challenge tests or in conditions of natural exposure [10, 23, 24, 26–28]. On the other hand, some researchers did not observe an increased level of ICAM-1 and VCAM-1 in serum of patients with allergic rhinitis [25, 29]. In the present study, the plasmatic levels of ICAM-1 and VCAM-1 were significantly increased in patients with PAR compared to healthy volunteers (*p* < 0.001 and *p* < 0.001, resp.), with no differences between the groups. These results confirm previous published data, reflecting a systemic inflammation in patients with PAR.

The kinetics of VCAM-1 and ICAM-1 is different. In patients with AR, ICAM-1 is increased in nasal secretion in both perennial and seasonal AR from the period of onset [26]. On the other hand, the expression of VCAM-1 is upregulated in the nasal mucosa of patients with AR [28, 30], especially in the late phase of allergic response [31]. Our results are similar to those from the above aforementioned studies. Present research included patients with PAR under continuous natural exposure to allergens. The continuous exposure to indoor or/and outdoor allergens may explain a continuous production of mediators which promote eosinophil recruitments, explaining high plasmatic levels of ICAM-1 and VCAM-1.

Gorska-Ciebiada et al. [27] have shown that ICAM-1 values are significantly lower in patients with mild forms compared to those with moderate-severe rhinitis [27]. In the present study, there was no correlation between ICAM-1 and VCAM-1 and the severity of allergic rhinitis or type of sensitization. Another study showed that VCAM-1 and ICAM-1 grow during the pollen season and fall out during off-season [26]. In the present study, we did not investigate the kinetics of ICAM-1 and VCAM-1. Most of the patients have polysensitization to both indoor and outdoor allergens which may explain the high level of CAMs in serum, due to their continuous production.

Interestingly, E-selectin was similar in patients with AR and healthy volunteers. Similarly, Ural et al. found that the E-selectin value did not differ in patients with allergic and nonallergic rhinitis in nasal lavage fluid [23]. E selectin is involved in leukocyte orientation, and it is a light adhesion marker, while ICAM-1 is a leukocyte-binding adhesion marker. The basal level of E-selectin positively correlated with ICAM-1 (*R* = 0.353, *p* = 0.001). This observation might be explained by their involvement at the beginning of cell recruitments. In patients with AR, the level of E-selectin starts to increase within 15 hours after allergen exposure, [32] and it declines after 24 hours [23, 33]. These data may explain not only the low level of E-selectin in our patients but also the correlation between E-selectin and severity of symptoms (*R* = 0.996, *p* < 0.001), generated mainly by histamine release. As we mentioned before, we did not investigate the kinetics of CAMs in serum, and the patients were under natural exposure not after a controlled allergen challenge exposure. It might be interesting to investigate CAM and cytokine levels in patients with AR at different time points to analyze their kinetics in conditions of natural exposure to allergens.

IgE is the central molecule in the pathogenesis of allergic diseases. It increases after sensitization and binds to mast cells through specific receptors, but a soluble portion remains in the serum and can be determined. In different clinical studies, the IgE level did not correlate with ICAM-1 or TNF-*α* values, which are higher in asthmatics but not in those with AR [29]. In our study, there was a significant correlation between the Eo count and total IgE (*p* < 0.001), but the total IgE values did not correlate with other markers of inflammation, such as CAMs or FENO. Although CAMs are involved in Eo migration at the site of inflammation, the serum values of Eo did not correlate with ICAM-1 and VCAM-1 in our study. It is interesting to evaluate the level of CAMs in the nasal mucosa and to correlate with local infiltration of Eo, but in our study we did not perform such kind of investigation.

There is hypothesis suggesting that Eo recruited by CAMs can induce nitric oxide synthase in the epithelial cells of the bronchial mucosa. Nitric oxide (NO) in the exhaled air is known as the marker of eosinophilic inflammation in the lower respiratory tract. IgE-mediated inflammation results in elevated NO in the expired air [34, 35]. Studies have also shown that patients with AR during pollination have elevated NO levels in the air, even if their asthma symptoms are missing or mild [36]. Other studies showed elevated levels of exhaled NO and adenosine in patients with AR versus healthy subjects [34, 35], suggesting that a subclinical inflammation in the lower airways could exist in AR. In our group of patients, FeNO was increased in patients with AR and did not correlate with any of the studied markers. There was no correlation between the initial FeNO and the severity of rhinitis' symptoms, type of sensitization, or basal values of CAM (*p* > 0.05). But this lack of correlations cannot exclude a possible minimal inflammatory process in both the nasal and lower airway mucosa, other factors having an additional contribution to progression of inflammation in lower airways, like TNF-*α*-stimulation [37]. It might be interesting to monitor the evolution of patients in order to investigate if basal values of FeNo and CAMs could predict the occurrence of asthma after a period of time.

In this study, we also assessed the efficacy of H1 antihistamines, desloratadine and levocetirizine, in the therapy of AR. We also investigated a possible anti-inflammatory effect of both drugs, demonstrated by reduction of CAMs and FeNO. Several studies showed the efficacy of H1 antihistamines in allergic rhinitis [1, 2]. H1 antihistamines are now considered the first-line treatment in AR [1]. In our study, we observed that both desloratadine and levocetirizine improved nasal symptoms, reducing significantly TSS after 1 month of treatment, similar to previous published data [2, 19, 38]. TSS significantly decreased after treatment, with no differences between the investigated drugs (*p* = 0.571).

In our study, we evaluated the effect of desloratadine and levocetirizine on E-selectin, ICAM-1, and VCAM-1. After the four-week treatment, H1 antihistamines significantly decreased the plasmatic levels of ICAM-1 (*p* = 0.049) and E-selectin (*p* = 0.002), but not VCAM-1 (*p* = 0.310) compared to basal values. There was no difference between levocetirizine and desloratadine in reduction of CAM plasmatic levels. In the present study, we also noticed a significant reduction of CAM levels in patients with moderate-severe forms compared to patients with mild rhinitis (VCAM-1 *p* = 0.037, ICAM-1 *p* = 0.001 and E-selectin *p* = 0.002). But the reduction of CAM levels was not influenced by patients' age, sex, and type of sensitization.

*In vitro* studies demonstrated that not all 2nd-generation H1 antihistamines had anti-inflammatory effect. Cetirizine did not influence E-selectin, ICAM-1, and VCAM-1 *in vitro* studies although the authors noted an underexpression of ICAM-1 in epithelial cells of patients with AR treated with cetirizine [11]. Loratadine was seen to influence the level of VCAM-1 but not ICAM-1 in patients with monosensitization to house dust mites [12]. Regarding fexofenadine (an active metabolite of terfenadine), several studies showed its similar efficiency to cetirizine [39], loratadine [40], desloratadine, and levocetirizine [38] in improving rhinitis symptoms. But Schäper et al. [13] also demonstrated its anti-inflammatory effect, due to reduction of ICAM-1 in nasal secretions after 14 days of treatment [13]. *In vitro* studies revealed that levocetirizine inhibited ICAM-1 [15] and downregulated the activity of P-selectins [41] and the expression of VCAM-1 [42]. Desloratadine induced downregulation of ICAM-1 [43]. In most of these studies, the used concentrations of AH1 were higher than the therapeutic ones [41]. There are also *in vivo* studies that revealed the anti-inflammatory effect of H1 antihistamines. Both desloratadine and levocetirizine reduced ICAM-1 and nasal Eo [44], similar to present results. In the present study, VCAM-1 was not reduced by AH1, only ICAM-1 and E-selectin. Probably, the expression of ICAM-1 and E-selectin, markers of initial allergic response, is related to histamine release from mast cells, while other new synthesized cytokines and chemokines are probably involved in the expression of VCAM-1.

One-month evaluation revealed a significant decrease of IgE plasmatic level (*p* < 0.001) especially in patients with monosensitization either to indoor or outdoor allergens (*p* = 0.05). Total IgE significantly decreased in patients with moderate-severe forms of AR compared to those with mild disease (*p* = 0.05). The same pattern was also noticed also for peripheral Eo, with a significant reduction after treatment (*p* = 0.04). These results are similar to previous reported data [16, 44–46], for rupatadine, levocetirizine, and desloratadine, after 2–4 weeks of treatment.

The effect of H1 antihistamines on lower airway subclinical inflammation in patients with AR has been demonstrated in few studies [19]. *In vitro* studies have shown that NO synthase activity can be downregulated by H1 antihistamine therapy [47]. Animal studies have demonstrated that histamine released by mast cell plays an important role in the production of FeNO and in the enhancement of bronchial hyperreactivity [48]. *In vivo*, it has been demonstrated that levocetirizine lowers the FeNO values after 3 months of treatment in children with mite allergy [42]. In our study, we observed that FeNO was significantly reduced after 1-month treatment with H1 antihistamines, and desloratadine was more effective than levocetirizine. This observation could be explained by the differences between investigated subgroups in relation with patient sensitization. The group treated with levocetirizine had a lower basal value of FeNO than desloratadine group, so the room for improvement of FeNO was more limited. To establish the clear role of each H1 antihistamines in reducing FeNo level, further extensive studies are required. But, this observation might open a new strategy in limiting subclinical inflammation using AH1. The continuous treatment with H1 antihistamines in patients with PAR might reduce the occurrence of asthma, confirming the previous published data [42].

There are some limitations of this study. Firstly, a small number of patients were included in the study, sensitized to both indoor and outdoor allergens. The randomization of the patients took into account the treatment, not the type of sensitization, which may explain the differences between investigated subgroups, more patients sensitized to pollen being included in levocetirizine subgroup. The study has another limitation, the lack of information regarding different anti-inflammatory effects of H1 antihistamines according to allergen exposure. It might be interesting to analyze the effect of AH1 on CAMs and other related mediators (cytokines and chemokines) and to differentiate the results according to the type of sensitization.

The present study emphasized the anti-inflammatory role of H1 antihistamines from 2nd generation, demonstrated by reduction of CAM plasmatic levels in patients with PAR. The reduction of CAMs was noticed in the plasma not in the nasal mucosa, in conditions of natural continuous exposure to allergens. Also, the research investigates two H1 antihistamines from 2nd generation in order to establish if there are significant differences between them in improving both clinical symptoms and inflammatory parameters.

## 5. Conclusions

Patients with PAR have high serum levels of ICAM-1 and VCAM-1. FeNO as a marker of subclinical inflammation is increased in patients with PAR. H1 antihistamines improve allergic rhinitis symptoms and reduce the markers of inflammations after 1 month of treatment. Desloratadine has a better anti-inflammatory effect in reducing FeNO. Baseline values of CAMs did not predict the response to therapy.

## Figures and Tables

**Table 1:** Patients' demographic data.

Parameter	Desloratadine (*n* = 40)	Levocetirizine (*n* = 39)	*p*
Age^∗^	28.05 ± 6.32	32.89 ± 12.17	0.031
Sex^∧^			
Male	57.5% (23)	43.6% (17)	0.263
Female	42.5% (17)	56.4% (22)	
Living area^∧^			
Urban	85% (34)	82.1% (32)	0.770
Rural	15% (6)	17.9% (7)	
Allergic rhinitis onset (months)°	24 (6–60)	36 (7.5–68)	0.532
Allergen sensitization^∧^			
Indoor	37.5% (15)	5.1% (2)	0.002
Outdoor	17.5% (7)	35.9% (14)	
Indoor + outdoor	45% (18)	59% (23)	
Severity^∧^			
Mild	25% (10)	33.3% (13)	0.465
Moderate-severe	75% (30)	66.7% (26)	

^∗^Data are expressed as mean ± SD; ^∧^data are expressed as %, *n*; °data are expressed as median, 25–75th percentile. SD: standard deviation; *n*: number.

**Table 2:** Plasmatic values of total IgE and adhesion molecules in healthy volunteers and patients with AR.

Parameter	Healthy volunteers (*n* = 30)	Patients with AR (*n* = 79)	*p*
Total IgE (UI/l)	<100	115 (45.3–169)	**<0.001**
ICAM-1 (ng/ml)	111.21 (100–206.30)	218.19 (189.13–266.65)	**0.001**
VCAM-1 (ng/ml)	557 (249–891)	1004.02 (822.32–1174.68)	**<0.001**
E-selectin (ng/ml)	32.03 (23.68–45.94)	33.81 (24.61–47.53)	0.404

Data are expressed as median, 25–75th percentile. Significance *p* < 0.05.

**Table 3:** Patients' biological parameters before and after treatment.

Parameter		Desloratadine (*n* = 40)	Levocetirizine (*n* = 39)	*p*
Total IgE	Baseline	116.5 (46.25–269)	115 (45.3–269)	0.212
	4 weeks	65 (28.32–167.5)	75 (30–150)	

Eo	Baseline	5.00 (3.20–6.50)	5.20 (2.70–7.80)	0.04
	4 weeks	4.10 (2.60–5.80)	4 (2.35–6.35)	

VCAM-1	Baseline	1037.8 (878.19–1200.82)	919.32 (818.5–1136.02)	0.202
	4 weeks	1037.98 (897.64–1193.09)	913.56 (703.58–1128.60)	

ICAM-1	Baseline	208.12 (179.95–259.04)	229.81 (195.75–275.21)	0.355
	4 weeks	205.58 (170.93–256.01)	206.13 (182.74–270.14)	

E-selectin	Baseline	33.54 (25.72–46.57)	33.81 (23.95–50)	0.459
	4 weeks	33.07 (24.46–44.89)	31.90 (22.08–49.5)	

FeNO	Baseline	38 (19–49)	23 (16.25–43)	0.05
	4 weeks	14 (11–21)	17.5 (14–22.5)	

Data are expressed as median, 25–75th percentile. Significance *p* < 0.05.

## Data Availability

The data used to support the findings of this study are available from the corresponding author upon request.
